# Contextualising facial expressions: The effect of temporal context and individual differences on classification

**DOI:** 10.1177/17470218221094296

**Published:** 2022-06-06

**Authors:** Kinenoita Irwantoro, Nathali Nimsha Nilakshi Lennon, Isabelle Mareschal, Ahamed Miflah Hussain Ismail

**Affiliations:** 1 School of Psychology, University of Nottingham Malaysia, Semenyih, Malaysia; 2 School of Biological and Behavioural Sciences, Queen Mary University of London, London, UK

**Keywords:** Facial expressions, contextual effects, temporal context, individual differences

## Abstract

The influence of context on facial expression classification is most often investigated using simple cues in static faces portraying basic expressions with a fixed emotional intensity. We examined (1) whether a perceptually rich, dynamic audiovisual context, presented in the form of movie clips (to achieve closer resemblance to real life), affected the subsequent classification of dynamic basic (happy) and non-basic (sarcastic) facial expressions and (2) whether people’s susceptibility to contextual cues was related to their ability to classify facial expressions viewed in isolation. Participants classified facial expressions—gradually progressing from neutral to happy/sarcastic in increasing intensity—that followed movie clips. Classification was relatively more accurate and faster when the preceding context predicted the upcoming expression, compared with when the context did not. Speeded classifications suggested that predictive contexts reduced the emotional intensity required to be accurately classified. More importantly, we show for the first time that participants’ accuracy in classifying expressions without an informative context correlated with the magnitude of the contextual effects experienced by them—poor classifiers of isolated expressions were more susceptible to a predictive context. Our findings support the emerging view that contextual cues and individual differences must be considered when explaining mechanisms underlying facial expression classification.

## Introduction

Humans use facial expressions as a form of non-verbal communication. Classification of facial expressions allows us to infer the expresser’s underlying emotional state. It is a universal ability of people across different cultures, at least when it comes to expressions conventionally referred to as “basic,” such as happy and sad (Ekman & Friesen, 1971; Tracy & Randles, 2011). In real-life scenarios, facial expressions almost always occur within a meaningful context. This context is both spatial (it appears together with the face) and temporal (the context and face separated in time). As discussed below, contextual cues can alter the perceived category and/or affect the classification speed of facial expressions that are otherwise classifiable on their own.

Some contextual cues originate from the expresser. For basic expressions, when the expresser’s body gestures are congruent in affect with the face, they lead to more accurate classifications of the facial expression (Aviezer et al., 2008; Meeren et al., 2005) and when the expresser’s vocal tones are congruent in affect, they make classification faster (Dolan et al., 2001). Some contextual cues are external, arising outside of the expresser. For example, expressions of individuals seen with or before the expresser can bias classification (e.g., make a neutral face look happier; Gray et al., 2017; Liberman et al., 2018) or expedite classification (Werheid et al., 2005). Emotional congruency of static affective scenes (e.g., a picture of a road accident) can also categorically alter perceived facial expressions, when they appear simultaneously with the face (Righart & de Gelder, 2008) or precede the face (Hietanen & Astikainen, 2013). The famous Kuleshov Effect is another related example. Here, objects in a scene (e.g., a coffin) can bias the classification of a facial expression (e.g., a neutral face appears sad), as long as an interaction between the expresser and the object is implied. This effect occurs irrespective of whether the object is shown as a static picture or a short, dynamic (3 or 6 s long) clip (Barratt et al., 2016; Calbi et al., 2017). Furthermore, classification of posed expressions as well as candid expressions taken from real-world scenarios are also categorically altered by the knowledge of what has happened before someone makes an expression, when provided in the form of a story or short sentences describing a situation (Carroll & Russell, 1996; Diéguez-Risco et al., 2013; Kayyal et al., 2015). Finally, even the anticipation of an affective consequence from simple cues (e.g., anticipating a shock from a colour cue) facilitates the classification of facial expressions congruent with the anticipated affect (Kavcıoğlu et al., 2021).

It must be noted that the extent of the effect from some external contextual cues seems to depend on characteristics of the perceiver too, e.g., the perceiver’s age. While congruent body cues from an expresser facilitate the classification of their facial expression compared with incongruent body cues, this advantage is more pronounced in older adults who are worse than younger adults in classifying isolated facial expressions (Noh & Isaacowitz, 2013). This may reflect older adults’ greater reliance on context to compensate for their decline in classification ability (Abo Foul et al., 2018). Culture appears to play an important role too. Japanese participants’ classification of facial expressions was biased by the emotion conveyed by spatial contextual faces surrounding the test face, but this was not the case for American participants, a result the authors attribute to cultural differences in allocating attention to the context (Masuda et al., 2008). Behavioural tendencies to process affective information could be another factor. Lee et al. (2012) measured these tendencies using self-report scales and showed that increased tendencies to process positive information made people more susceptible to positive contexts (i.e., spatial affective scenes), whereas increased tendencies to process negative information made people more susceptible to negative contexts.

Although age, attention, and behavioural tendencies seem to modulate contextual effects, whether individual differences in younger adults’ ability to classify facial expressions on their own affects how susceptible they are to contextual cues remains largely unexplored. In a review by Aviezer et al. (2017), the authors present some re-analysed data from earlier studies and state that viewers’ susceptibility to context was not related to their ability to classify facial expressions appearing alone. However, these studies only used static images (e.g., body cues) as spatial context. Furthermore, Yamashita et al. (2016) measured participants’ ability to identify and describe emotions using a self-report scale and correlated it with the magnitude of the contextual effects experienced when detecting shifts in facial expressions. They found that the susceptibility to contexts increased as a function of increasing self-reported abilities, when detecting subtle, but not large, shifts in expressions. However, self-reported abilities did not correlate with participant’s accuracy to detect shifts in expressions without a meaningful context, suggesting that self-reports may not accurately reflect individual differences in face recognition per se. To gain better insight into individual differences, it is important to obtain behavioural measures of individual differences in classifying facial expressions in isolation and examine its relationship with susceptibility to context.

Most studies reported above isolate contextual cues, measuring their effects one at a time. However, in real life, a facial expression is often encountered while experiencing a more perceptually rich scenario that dynamically unfolds over time. These perceptual experiences often combine visual and/or auditory cues, as well as somatosensory cues such as touch (e.g., a friend holding your hand in grief). To our knowledge, only Chen and Whitney (2019) used a dynamic and naturalistic context that closely resembled natural experiences in tasks where a visually blurred character’s emotional state needed to be inferred. Chen and Whitney’s (2019) findings are specific to the effect of spatial context on evaluating the emotion of the individual (i.e., not just the facial expressions per se) along two primary dimensions of emotions, valence, and arousal. We also experience scenarios where the context precedes the facial expression in time, and whether a dynamic and naturalistic temporal context would influence the categorical perception of facial expressions is still unclear. Although studies examining the Kuleshov Effect (Barratt et al., 2016; Calbi et al., 2017) have indeed demonstrated the effect of dynamic, albeit brief, temporal contexts on evaluating an expression dimensionally (e.g., for their valence) as well as categorically (e.g., as happy), these effects are specific to static neutral faces. Whether such effects generalise to other basic expressions or even more interestingly to those that are not conventionally referred to as “basic” remains unknown. Indeed, contextual effects are known to be relatively stronger for non-basic expressions such as pain and hope (Carroll & Russell, 1996; see above) and when the expression is ambiguous (Lee et al., 2012).

Accordingly, here we aimed to determine (1) whether temporal context, provided in the form of a dynamic, perceptually rich and naturalistic experience, would influence the classification of subsequently encountered basic and non-basic facial expressions and (2) whether people’s ability to classify facial expressions without any cues is related to their susceptibility to contextual cues. Following a similar design to Chen and Whitney (2019), we presented participants with movie clips as the temporal context. Movie clips are good proxies for real life as they are staged to emulate real scenarios. One of the character’s (the “expresser”) face in each movie clip was masked, while the rest of the expresser’s body was visible, as were other interacting individuals and background scenes. Participants could also hear the expresser and other characters speaking, as well as background sounds such as the noise from a moving vehicle. After watching movie clips, participants were asked to classify a facial expression that was unfolding from neutral to either reflect genuine happiness (happy smile) or sarcasm (sarcastic smile). We chose sarcastic expressions because they are a non-basic emotion but share some perceptual similarity with happy expressions (Gutiérrez-García & Calvo, 2015), and contextual effects are stronger when this is the case, although this has only been demonstrated for basic expressions (Aviezer et al., 2008). We used dynamic faces created using image-based morphing as stimuli to provide a closer approximation of naturally encountered unfolding expressions and offer some control over the intensity of the expression (Dobs et al., 2018). In these unfolding faces, the intensity increases over time. Therefore, the time it takes to classify the expression reflects how much expression intensity (i.e., expressiveness) is required to support accurate classification.

To examine the effect of temporal context, we manipulated the congruency between movie clips and faces by presenting clips that represented a happy or an angry scenario. We considered that happy clips would be congruent, and angry clips would be incongruent, with happy faces, and that angry clips would be congruent, and happy clips would be incongruent, with sarcastic faces. We assumed that angry movies (with a few sarcastically spoken statements in some clips) are more likely to lead to a final sarcastic expression, because sarcasm is known to be closely associated with anger (Bryant & Fox Tree, 2005), and negative contexts such as those invoking anger are known to facilitate the interpretation of sarcasm, although specific to spoken statements (Woodland & Voyer, 2011). We hypothesised that congruent (as compared with incongruent) contexts would increase the accuracy and reduce the speed of classifying both happy and sarcastic expressions. Changes in accuracy and speed of classification were taken as indices of the strength of the contextual effect. Accordingly, we also hypothesised that the strength of the contextual effect would be higher when participants are poorer at classifying facial expressions viewed without an informative context.

## Methods

### Participants

Fifteen participants (12 females; 20–27 years of age) were recruited to validate the movie clips (see below) and a separate cohort of 27 participants (18 females; 18–25 years of age) was recruited for the main experiment. All our participants were undergraduate students from the University of Nottingham Malaysia and participated for course credits. Informed consent (written) was obtained from all participants and experimental procedures were approved by the Science and Engineering Research Ethics Committee of University of Nottingham Malaysia (KI251019).

### Stimuli and apparatus

#### Context videos

Movie clips to be used as context videos were taken from TV shows and documentaries. Based on first impressions of the experimenters, they were divided into two types of clips: (1) those that represent a “happy” scenario (24 clips) and (2) those that represent an “angry” scenario (24 clips). We then ran these clips through a validation study to ensure that viewers identified happy clips as happy and angry clips as angry. Participants (*n* = 15) watched all 48 clips and after each one, classified it as either happy or angry in a two-alternative forced-choice task. All clips that achieved at least 80% accurate classifications (i.e., a minimum of 12 out 15 participants correctly classifying) were retained for the main experiment. Forty-four clips met this criterion and we used the first 40 of those to create the context videos (20 happy and 20 angry). All clips were in colour and all video frames within a clip had an identical dimension of 1280 × 720 pixels. The selected clips had a mean duration of 27.25 s (*SD* = 4.82 s; range = 24–34 s). There is some variability in the duration because we carefully cropped movies to ensure that a sensibly portrayed scenario ends in a climactic expression (i.e., with the camera focused on an identified main character’s face). This had the added benefit of preventing participants from predicting when the dynamic expression would appear, making them pay attention.

**Figure 1.**
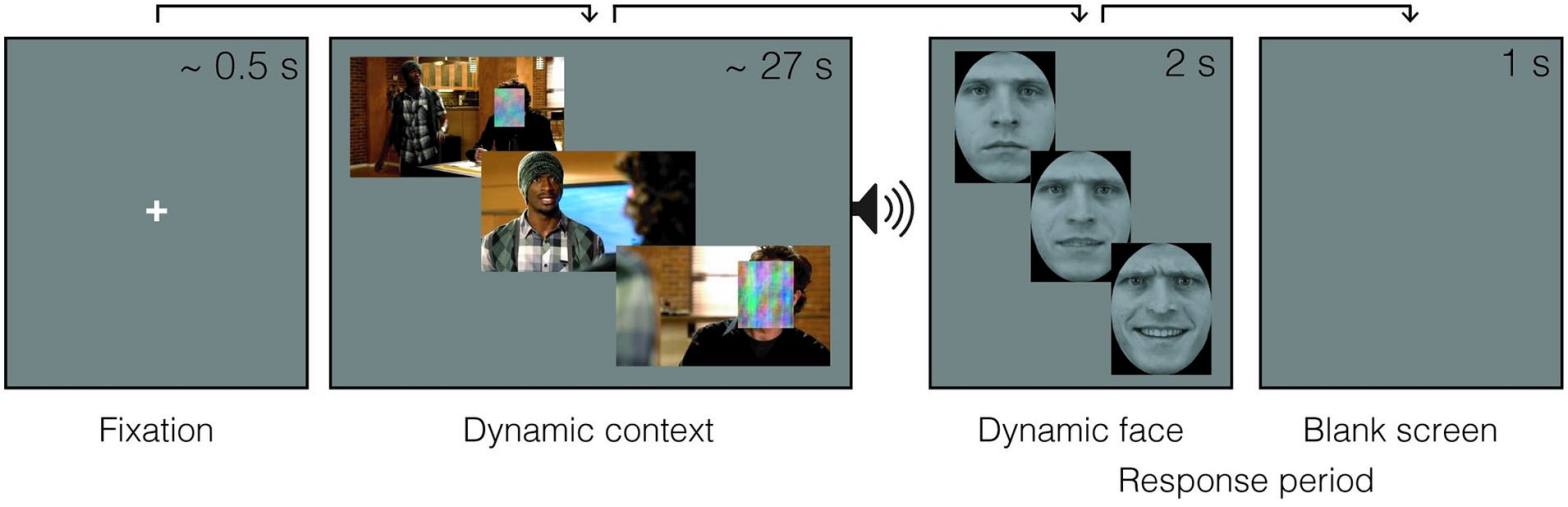
Timeline of a trial where a dynamic context is followed by a sarcastic facial expression. The video frames and morphed faces are arranged here to imply its dynamic nature. In the experiment, they were both centred on the screen and unfolded over time.

To create context videos, we first identified a single main character whose face would be masked in each clip. The last frame of each clip ended with the masked character’s face that remained masked. To mask the face, we marked the coordinates of the face on each video frame, whenever the face was within the frame and was not obscured by other characters. We inserted pixel noise (a uniform distribution of pixel values between 0 and 255 in all three colour channels) within these coordinates to mask the face on a frame-by-frame basis (Figure 1) and retained the audio for all frames. When the face was partially obscured, the mask size was changed to cover whatever regions were visible. When the face was fully obscured, no mask was used. We also combined a 27-s-long video purely made of pixel noise with an auditory white noise stimulus to create a “noise-context” video. Each video frame in it had a dimension of 1280 × 720 pixels and was created using a uniquely generated distribution of RGB pixels (uniformly distributed values between 0 and 255).

#### Morphed dynamic faces

Images of 20 different-identity Caucasian faces were obtained from the Karolinska Directed Emotional Faces Database (Lundqvist et al., 1998). Two Hispanic and two African American face identities were also obtained from the Racially Diverse Affective Expression (RADIATE) database (Conley et al., 2018). We chose faces from different races to match them to the masked characters’ race in the context videos. For all 24 identities, we gathered images of their faces with a neutral, happy, and angry expression, from the same databases. The resulting 72 images were converted to greyscale, cropped to remove all external facial features (e.g., hair, neck) and were resized to have 290 pixels in width and 380 pixels in height. To generate a sarcastic expression for each identity, we followed the procedure used by Gutiérrez-García and Calvo (2015) and blended the top half of an angry expression from a given identity with the bottom half of a happy expression from the same identity. The resulting blended faces contained facial markers such as lowered eyebrows (from angry faces) and smiling mouths (from happy faces) that are characteristic of sarcastic expressions (Attardo et al., 2003). All happy and sarcastic expressions used in the experiment are provided in Supplementary material Table S1.

We created two types of dynamic expressions by morphing images of distinct expressions, using custom written codes in MATLAB 2019a (MathWorks). Dynamic happy expressions were created by morphing a neutral expression of a given identity with its happy expression. Dynamic sarcastic expressions were created by morphing a neutral expression with a sarcastic expression of the same identity. Each dynamic expression contained a sequence of 120 morphed faces that gradually unfolded from neutral to happy or sarcastic. The 120 morphs from each sequence were combined to generate a 2-s-long video showing 60 morphed faces per second. Accordingly, dynamic happy and sarcastic expressions were created for each of the 24 identities.

#### Apparatus

All stimuli were displayed on an HP desktop monitor with a spatial resolution of 1600 × 900 pixels and a refresh rate of 60 Hz. Sounds in the context and noise-context videos were delivered through a Sennheiser PX-200 headphone. Image editing and video generation were achieved using Adobe Photoshop, iMovie and MATLAB. A programme written using PsychoPy (version 3.1.2) (Peirce et al., 2019) controlled the sequence and display parameters of stimulus presentation and recorded participants’ responses and response times.

### Procedure

Participants were asked to watch and listen to a series of videos and then classify facial expressions that immediately followed the videos. They were told that in some videos (i.e., happy or angry context videos), one character’s face would be masked, while in others (i.e., noise-context), the whole video would be masked. They were also told that the face that appears after the video belongs to the masked character’s face. After receiving instructions, each participant completed 60 experimental trials (Figure 1). Each trial started with a white fixation cross presented at the centre of the screen for 0.5 s. This was followed by a context or a noise-context video presented for its entirety. Participants then heard a beep sound for 0.5 s to mark the end of the video. Immediately after the offset of the beep sound, a dynamic expression was presented for 2 s, followed by a grey blank screen shown for 1 s. The participants were instructed to classify the dynamic expression as “happy” or “sarcastic,” by pressing the key “z” or “m” in the keyboard, respectively. Participants could respond anytime from the beginning of the dynamic expression to the end of the blank screen that followed it. They were asked to respond as quickly and as accurately as possible. After classifying, they were asked to rate the context video they watched in a given trial for familiarity (rating was not required for trials with a noise context). On a 4-point scale that was visible on the screen until response, they indicated whether the context was “Unfamiliar,” “Somewhat unfamiliar,” “Somewhat familiar,” or “Familiar,” by clicking on the corresponding label.

Experimental trials were divided into six conditions as characterised by the type of the context (happy context, angry context, and noise context) and the dynamic expression (happy and sarcastic). Twenty trials were presented for each type of context—happy, sarcastic, and noise context. Within the 20 trials, half the trials (10) contained a happy dynamic expression while the remaining (10) contained a sarcastic dynamic expression. The identities of the dynamic expressions were carefully chosen to match the race and gender of the masked faces in each context video. Some identities were used more than once (a maximum of three repeats within the course of the experiment; see Supplementary material Table S3), as long as they matched the masked character in race and gender. All trial types were randomised and presented within a single experimental block.

## Results

First, we excluded trials in which the participants did not manage to provide any response within the 3-s interval. Accordingly, a total of eight trials were excluded across all participants and all six experimental conditions; not more than two trials per participant and not more than one trial per experimental condition were excluded. Following this, we calculated the accuracy of correctly classifying the dynamic expressions as a proportion of all the remaining trials, separately for each participant. One of the participant’s mean accuracy for classification across all experimental trials was only 0.6. As it was very close to guessing (i.e., 95% bootstrapped confidence intervals overlapping with a guessing accuracy of 0.5; see Supplementary material Table S4) and was outside 2 standard deviations from the mean accuracy across all participants, we considered it an outlier and removed it from further analysis. Data from two other participants were also removed, as an unexpected power failure prevented completion of the experiment. Accordingly, data from 24 participants were included in the analyses reported below. For these participants, the mean accuracy was 0.83 (*SD* = 0.09).

Before proceeding into any further analyses, we wanted to confirm that most videos we presented were not familiar to our participants, because familiarity may help them anticipate facial expressions. We quantified the number of trials in which the participants indicated that the context videos were “familiar” or “somewhat familiar” (see Supplementary material Table S2). On average, 16% of context videos were recognised across all participants (range = 0%–53%). When we quantified it by condition, we obtained the following percentages: happy context-happy expression = 15%, happy context-sarcastic expression = 10%, angry context-happy expression = 15%, and angry context-sarcastic expression = 25%. To summarise, it is clear that a large percentage of context videos were not familiar to our participants (more details provided in Supplementary material Table S2).

**Figure 2.**
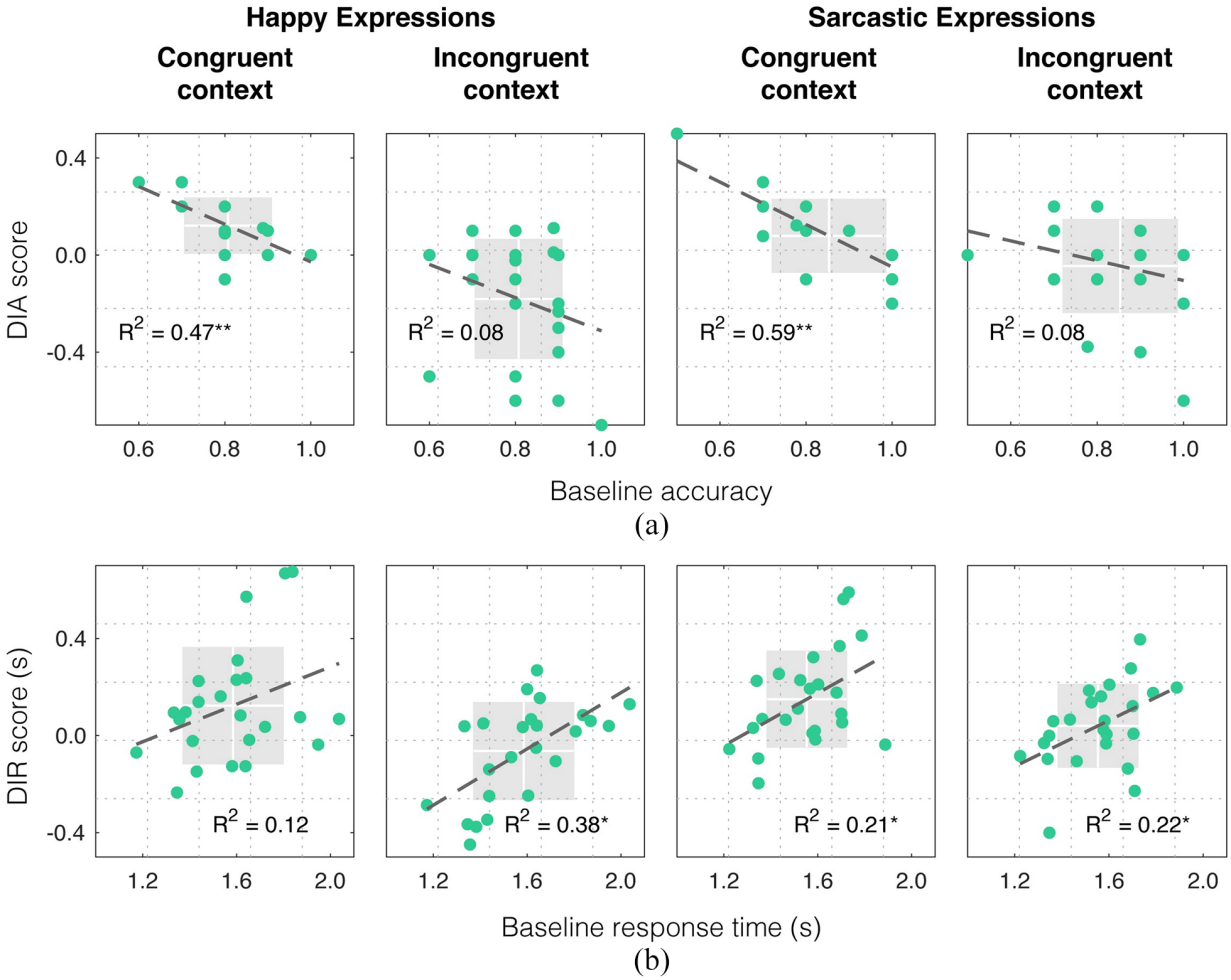
Contextual effects for happy and sarcastic facial expressions. (a) DIA scores (green circles) plotted against baseline accuracy, for each expression, with congruent and incongruent contexts. (b) DIR scores (green circles) plotted against baseline response time, for each expression, with congruent and incongruent contexts. In all subplots, thicker grey dashed lines represent least-squares fit to the data. Vertical and horizontal lines in white denote the mean for data plotted along the x and y axes, respectively. Surrounding grey shaded regions denote ± 1 standard deviation around the mean, for means plotted along the x and y axes. *R*^2^ values correspond to the square of Pearson’s correlation coefficients. Asterisks denote significant correlations at the level of *p* < .05 (*) or *p* < .001 (**).

Following this, we calculated the mean accuracy of classification for each of the six experimental conditions, separately for every single participant. Similarly, we also calculated the mean response time to accurately classify expressions (i.e., response times for correct responses only). A summary of mean accuracies and response times across all participants is provided in Table 1. Individual means were used to calculate the magnitude of the contextual effect for each participant, by obtaining the “difference in accuracy” (DIA) and the “difference in response time” (DIR). To measure the DIA for a congruent context, we subtracted the mean accuracy in noise-context trials from the mean accuracy in trials with a congruent context, for the same dynamic expression (e.g., *happy context with happy expression - noise context with happy expression*). The DIA for an incongruent context was calculated by subtracting the mean accuracy in noise-context trials from the mean accuracy in trials with an incongruent context, for the same dynamic expression (e.g., *happy context with sarcastic expression - noise context with sarcastic expression*). DIR for congruent and incongruent contexts were calculated similarly for both happy and sarcastic expressions, but the resulting values were multiplied by –1 to facilitate interpretation. Accordingly, positive DIA and positive DIR values both indicate facilitated classification due to the context, whereas negative values indicate an impairment in classification due to the context. Figure 2 provides a summary of these values.

**Table 1.** Mean accuracy and response times across all participants for each of the six experimental conditions as characterised by the type of context and dynamic facial expression.

Type of context	Dynamic facial expression	Accuracy ( ±1 standard deviation)	Response time ( ±1 standard deviation) (s)
Happy	Happy	0.93 (0.09)	1.46 (0.26)
	Sarcastic	0.81 (0.20)	1.51 (0.18)
Angry	Happy	0.63 (0.24)	1.65 (0.18)
	Sarcastic	0.93 (0.10)	1.40 (0.19)
Noise	Happy	0.81 (0.10)	1.59 (0.21)
	Sarcastic	0.85 (0.13)	1.55 (0.17)

Mean DIA values (Figure 2) were subjected to a 2 × 2 repeated-measures analysis of variance (ANOVA), with dynamic expression (“happy” and “sarcastic”) and congruency (“congruent” and “incongruent”) as within-subjects factors. There was no main effect of dynamic expression, $F(1,23)=1.90,p=.182,\eta_{p}^{2}=.076$ , showing that contextual effects, if any, influenced the classification accuracy for both expressions in a similar manner. However, a significant main effect of congruency, $F(1,23)=32.62,p<.001,\eta_{p}^{2}=.586$ , indicated that a congruent context produced higher DIA scores than an incongruent context. The ANOVA also revealed a significant interaction between congruency and dynamic expression, $F(1,23)=18.28,p<.001,\eta_{p}^{2}=.443$ . Further analysing the interaction, Bonferroni-corrected two-way repeated measures paired samples *t*-tests revealed that an incongruent context impaired the classification accuracy for happy expressions significantly more than for sarcastic expressions, $t(23)=-3.37,p=.006,Cohen^{’}s\;\;d=-0.687$ . On the contrary, a congruent context did not differentially affect classification of happy or sarcastic expressions, $t(23)=1.07,p=.295,Cohen^{’}s\;\;d=0.219$ . We also performed an ANOVA on mean DIR values (Figure 2). There was no main effect of dynamic expression, $F(1,23)=2.56,p=.123,\eta_{p}^{2}=.100$ . There was a main effect of congruency, $F(1,23)=14.40,p<.001,\eta_{p}^{2}=.385$ , indicating that a congruent context led to faster response times than an incongruent context. As revealed by the ANOVA, there was no significant interaction between congruency and dynamic expression, $F(1,23)=2.50,p=.127,\eta_{p}^{2}=.098$ .

Mean DIA values in Figure 2 indicate that, compared to when a noise context was provided, classifications were more accurate with congruent contexts and less accurate (i.e., impaired) with incongruent contexts. To verify this, we ran separate two-tailed one-sample *t*-tests to compare mean DIA for congruent and incongruent contexts (irrespective of expression) against zero. We found that congruent contexts significantly facilitated classification, $t(23)=5.31,p<.001,Cohen^{’}s\;\;d=1.085$ , whereas incongruent contexts significantly impaired classification, $t(23)=-2.81,p=.010,Cohen^{’}s\;\;d=-0.573$ . For response time data, separate two-way one-sample *t*-tests showed that congruent contexts (irrespective of expression) facilitated classification with faster response times, $t(23)=3.46,p=.004,Cohen^{’}s\;\;d=0.706$ , whereas incongruent contexts (irrespective of expression) neither facilitated nor impaired response times, $t(23)=-0.42,p=.678,Cohen^{’}s\;\;d=-0.086$ .

Next, we assessed the relationship between individual differences in our participants’ ability to classify facial expressions in isolation and the magnitude of the contextual effect they experienced, by running a series of Pearson’s product-moment correlation tests. We correlated our participants’ accuracy to classify an expression (happy or sarcastic) with a noise context (defined as the “baseline accuracy”) with their DIA scores associated with the same expression (Figure 2). When the context was congruent, DIA scores significantly correlated with participants’ baseline accuracies for both happy, r(22)=−.69,p<.001 , and sarcastic, r(22)=−.77,p<.001 , expressions. Correlations were not significant when the context was incongruent (p>.05) . We also correlated our participants’ response time to classify an expression (happy or sarcastic) with a noise context (defined as the “baseline response time”) with their DIR scores associated with the same expression (Figure 2). When the context was congruent, DIR scores significantly correlated with participants’ baseline response times for sarcastic expressions, r(22)=.46,p=.023 , but not with baseline response times for happy expressions, r(22)=.34,p=.100 . When the context was incongruent, DIR scores significantly correlated with participants’ baseline response times for happy expressions, r(22)=.62,p=.001 , as well as with baseline response times for sarcastic expressions, r(22)=.47,p=.021 .

## Discussion

Here, we examined people’s susceptibility to dynamic, audiovisual contextual cues when classifying dynamic basic (happy) and non-basic (sarcastic) facial expressions that briefly followed the context. We show that a congruent temporal context leads to more accurate and faster classifications of happy and sarcastic expressions, compared with when the same expressions are classified with an uninformative context (i.e., with a noise context). In contrast, when the same expressions were classified after experiencing an incongruent temporal context, classifications were less accurate but similar in speed, compared with classifications that followed a noise context. The lack of a cost in classification speed for incongruent contexts is not necessarily in contradiction with the literature showing slower classification for faces cued by incongruent contexts (Diéguez-Risco et al., 2013; Dolan et al., 2001; Hietanen & Astikainen, 2013; Meeren et al., 2005; Righart & de Gelder, 2008; Werheid et al., 2005). Previous studies often establish congruency effects by comparing response times between classification of expressions with a congruent and an incongruent context (Diéguez-Risco et al., 2013; Dolan et al., 2001; Hietanen & Astikainen, 2013; Meeren et al., 2005; Righart & de Gelder, 2008), and it is impossible to determine whether the effect is due to facilitation from a congruent context or impairment from an incongruent context. Therefore, it is important to compare classification with a context against a no context condition. By doing so, we demonstrate an asymmetry where a congruent temporal context improves classification speed whereas an incongruent context does not impair it.

Although past studies have demonstrated categorical shifts in perceived facial expressions caused by isolated temporal contextual cues (Carroll & Russell, 1996; Hietanen & Astikainen, 2013; Kavcıoğlu et al., 2021; Kayyal et al., 2015), they do not often capture the dynamic and perceptually rich contextual cues present in real life. Studies on the Kuleshov Effect have indeed utilised more realistic temporal contexts (i.e., brief dynamic scenes without audio), but their influence has only been demonstrated on neutral facial expressions, and that too only if the expresser’s attention towards cues in the background scene is clearly implied. We show that realistic dynamic temporal contexts cause categorical shifts in the categorisation of non-neutral expressions. However, we cannot determine whether our contextual effects were dependent on an implied interaction between the expresser and contextual cues (cf. Gray et al., 2017). Indeed, although all classified faces were directly gazing at the participant, knowing that they belonged to a character who was interacting with the context might have implied interaction. Moreover, even if interaction was implied, it is not clear whether these external cues had any influence at all, because our contexts also contained cues from the expresser (e.g., their body language). Isolating the contribution of different temporal cues is beyond the scope of this study but should be explored in the future.

In contrast to previous studies on contextual effects using static faces (except Israelashvili et al., 2019, who examined valence judgements only), we used dynamic faces as stimuli. The expressiveness of our faces increased from neutral to happy or sarcastic over 2 s, and participants’ response times were concentrated within this 2-s period. For four out of six experimental conditions, two standard deviations above their mean response time (i.e., 95% of response time data) fell within 2 s (Table 1). For the remaining two conditions, they were still within 2.01 s (Table 1). If we assume constant stimulus-response delays within participants, this implies that response times are proportional to the expressiveness of the dynamic faces. Therefore, faster responses due to congruent contexts also reflect participants’ ability to accurately classify basic and non-basic expressions with less intensity. Accordingly, we report novel findings showing that temporal contexts can also reduce the intensity of facial expressions required to be accurately classified.

We expected the contextual effect to be stronger for sarcastic expressions because they convey mixed emotional signals (i.e., happy mouth and angry eyes) and contextual effects are known to be greater for vague or complex expressions (Carroll & Russell, 1996; Kavcıoğlu et al., 2021; Lee et al., 2012). However, our findings suggested otherwise. While facilitation resulting from a congruent context was comparable between happy and sarcastic expressions, the impairment caused by an incongruent context was more pronounced for happy than sarcastic expression categorisation. We propose that the enhanced impairment for happy faces may be attributed to happy faces being perceived more negatively when participants anticipate an aversive event (e.g., a threat of shock; Kavcıoğlu et al., 2021) or are induced with a negative emotional state (e.g., pain; Gerdes et al., 2012). Either of these effects may have occurred from experiencing an angry context by our participants (i.e., in addition to effects due to affective incongruency between context and face). This would result in happy expressions being more likely to be misclassified as sarcastic, an expression partly concealing negative valence emotions. Interestingly, these effects do not make participants perceive negative valence expressions (e.g., angry) as more negative (Gerdes et al., 2012; Kavcıoğlu et al., 2021), which could explain comparable levels of facilitation from congruent contexts between happy and sarcastic expressions.

One challenge in interpreting our findings stems from the definition of a “congruent” context. Although a happy context is fully congruent with a happy expression, the affective congruency of angry contexts with sarcastic facial expressions is less clear. A sarcastic expression’s top half is congruent with an angry context, but its bottom half is congruent with a happy context. Despite this complexity, angry contexts produced more accurate and faster classification of sarcastic expressions, compared with happy contexts. In fact, the magnitude of this facilitation was comparable to the facilitation resulting from a happy context on happy expressions. In that case, the facilitated classification of sarcastic expressions with angry contexts could reflect the fact that angry contexts were more likely to predict a sarcastic ending, rather than a happy context (Woodland & Voyer, 2011). This suggests a distinction between a “predictive context” and a “congruent context.” Previous studies on congruency effects didn’t have to be concerned about this distinction because the two always coincided. Nonetheless, our findings raise questions about whether context-based predictions can alter facial expression classification beyond affective congruency. For now, we would settle with the interpretation that a predictive context facilitates the classification of an upcoming facial expression.

Finally, we show for the first time that the magnitude of the contextual effects we observed was strongly related to our participants’ ability to classify isolated facial expressions. As far as accuracy is concerned, this relationship was only true for facilitation resulting from a congruent context. For participants who were poorer at accurately classifying isolated expressions, a congruent context led to a greater improvement in accuracy than for participants who were more accurate at classifying isolated expressions. Our finding does not align with the re-analysed data presented in Aviezer et al.’s (2017) review. While the studies they re-analysed used static backgrounds as spatial context, we presented a perceptually rich, dynamic temporal context. Therefore, the nature and the timing of context, as well as the context’s and the classified face’s close resemblance to real-life experiences, could be important in elucidating individual differences.

Our findings are consistent with some previous studies (Lee et al., 2012; Masuda et al., 2008; Noh & Isaacowitz, 2013), in showing that susceptibility to contextual effects is not uniform across individuals. We show that there is a clear variability in susceptibility to context between young adults. In our case, participants who had difficulties in classifying isolated expressions (i.e., poor accuracy) were more reliant on context. Whether this difficulty contributes directly to the variability in susceptibility or if it is mediated by factors such as distinct strategies of attention deployment during classification (e.g., Masuda et al., 2008) is unclear. Consistent with Noh and Isaacowitz (2013), we also show that people who have difficulties classifying facial expressions may compensate by using the context more. The same could apply to clinical populations with known deficits in facial expression classification such as Prosopagnosics (Biotti & Cook, 2016) and those with autism spectrum disorder (Loth et al., 2018). It must be noted that our participants included in the analyses generally had above chance classification accuracy, especially when no context was provided. Therefore, it is important to consider individual differences within non-clinical populations too, when examining contextual effects.

For contextual effects on classification speed, the relationship with individual differences was rather complex. When classifying happy and sarcastic expressions, an incongruent context resulted in smaller impairments in response times for participants who were slower at accurately classifying isolated expressions than for participants who were quicker at accurately classifying isolated expressions. No clear relationship was observed for effects arising from a congruent context on happy expressions. Furthermore, when classifying sarcastic expressions, for participants who were slower at accurately classifying isolated expressions, a congruent context resulted in smaller response time facilitation than for participants who were quicker at accurately classifying isolated expressions. Overall, the relationship between congruency effects on classification speed and individual differences in classifying expressions without an informative context seems to depend on the type of expression (basic or non-basic) and the congruency of the contextual cue. More research is needed to examine what is driving these interactive and complex effects.

## Conclusion

Consistent with previous demonstrations of contextual effects, our findings challenge the idea that categorical perception of facial expressions is determined primarily by facial attributes. Contextual information appears to cause categorical shifts in the judgement of facial expressions. Facial expressions are therefore not limited to a perceptual classification of emotion, but rather extend to cognition where classification is a product of the faces being evaluated against a context or situation (Brosch et al., 2010). This is in favour of appraisal theories of emotion, which suggest that expressions of emotions are a product of the expresser’s evaluation of the internal and external circumstances leading to it (Ellsworth & Scherer, 2003). If the observer can infer this evaluation from perceivable cues in the context, it could guide the observer’s judgement of the facial expression (cf. Sander et al., 2007). However, whether effortful appraisals are required for contextual effects to occur is still unclear (Hassin et al., 2013). Given the emerging literature on susceptibility of facial expressions to context, it is important to start formulating theories and models that can explain the mechanisms underlying the integration of extrinsic cues and intrinsic characteristics (i.e., individual differences) during the classification of basic and non-basic facial expressions. According to Wieser and Brosch (2012), models that emphasise on distributed neural networks involved in face perception might be a good starting point.

## Supplemental Material

sj-docx-1-qjp-10.1177_17470218221094296 – Supplemental material for Contextualising facial expressions: The effect of temporal context and individual differences on classification Click here for additional data file. Supplemental material, sj-docx-1-qjp-10.1177_17470218221094296 for Contextualising facial expressions: The effect of temporal context and individual differences on classification by Kinenoita Irwantoro, Nathali Nimsha Nilakshi Lennon, Isabelle Mareschal and Ahamed Miflah Hussain Ismail in Quarterly Journal of Experimental Psychology
