# Supplementary material for: Contextualising facial expressions: The effect of temporal context and individual differences on classification
Source: Q J Exp Psychol (Hove). 2022 Jun 6;76(2):450–9. doi: 10.1177/17470218221094296 (PMC9896254; doi:10.1177/17470218221094296)
Supplement: sj-docx-1-qjp-10.1177_17470218221094296 – Supplemental material for Contextualising facial expressions: The effect of temporal context and individual differences on classification [file sj-docx-1-qjp-10.1177_17470218221094296.docx]

Supplementary Material for:

**Contextualising facial expressions: The effect of temporal context and individual differences on classification**

Kinenoita Irwantoro, Nathali Nimsha Nilakshi Lennon, Isabelle Mareschal, Ahamed Miflah Hussain Ismail

**Table S1.**

Happy and Sarcastic facial expressions (100% intensity) used in the experiment to create morphed dynamic facial expressions.

CAUCASIAN

| Identity | Happy Expression | Sarcastic Expression |
| --- | --- | --- |
| AF01 | 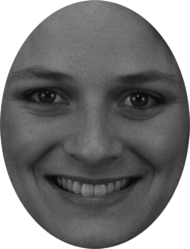 | 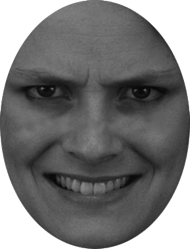 |
| AF06 | 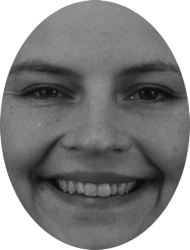 | 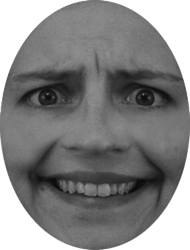 |
| AF09 | 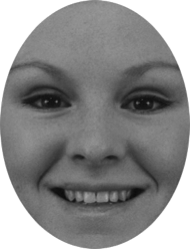 | 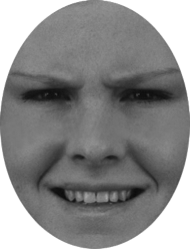 |
| AF14 | 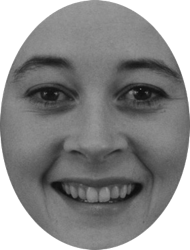 | 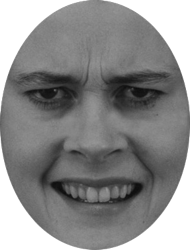 |
| AF16 | 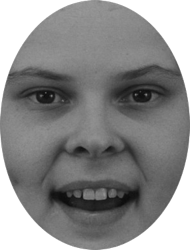 | 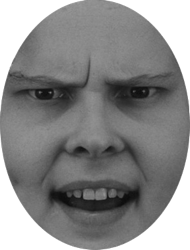 |
| AF26 | 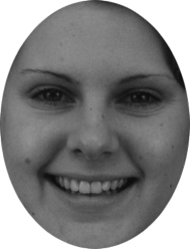 | 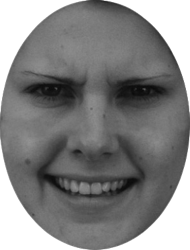 |
| AF27 | 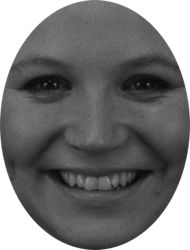 | 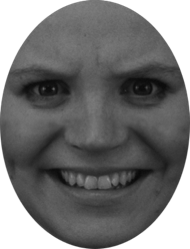 |

| BF22 | 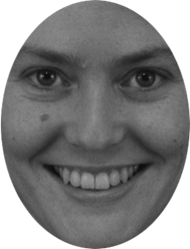 | 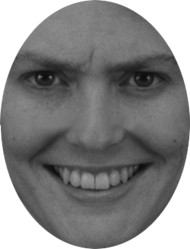 |
| --- | --- | --- |
| BF29 | 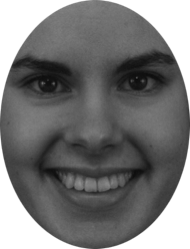 | 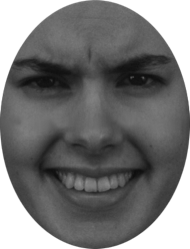 |
| BF32 | 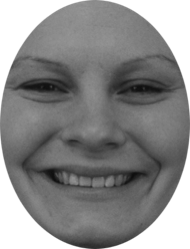 | 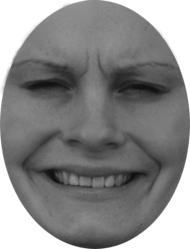 |
| AM07 | 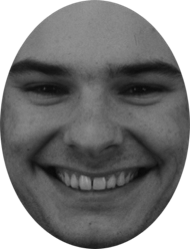 | 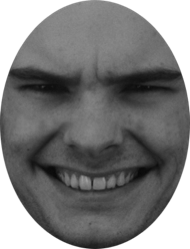 |
| AM23 | 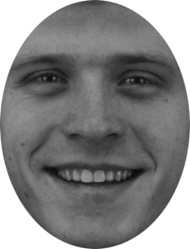 | 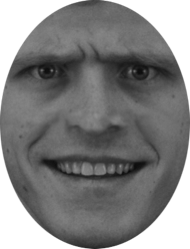 |
| BM04 | 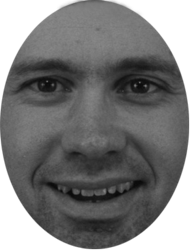 | 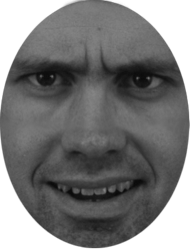 |
| BM05 | 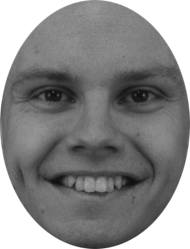 | 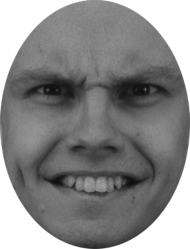 |

| BM11 | 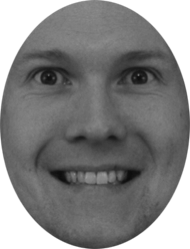 | 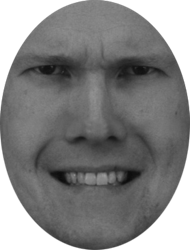 |
| --- | --- | --- |
| BM14 | 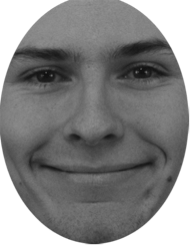 | 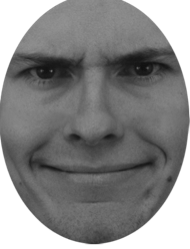 |
| BF21 | 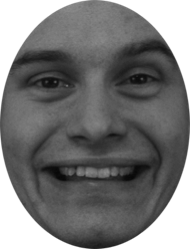 | 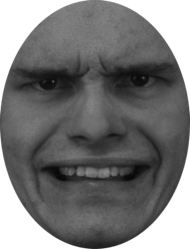 |
| BM31 | 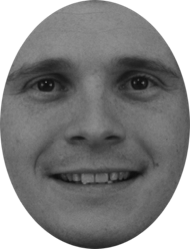 | 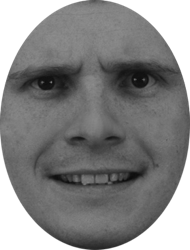 |
| BM34 | 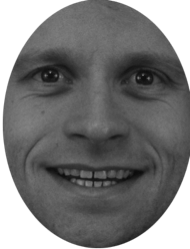 | 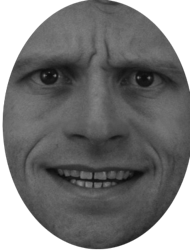 |
| BM35 | 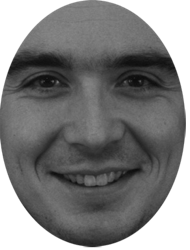 | 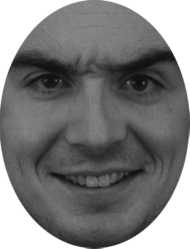 |

OTHER RACES

| Identity | Happy Expression | Sarcastic Expression |
| --- | --- | --- |
| AAF05 | 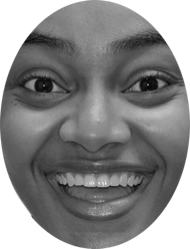 | 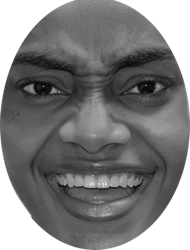 |
| HF01 | 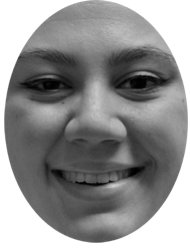 | 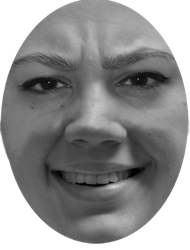 |
| AAM09 | 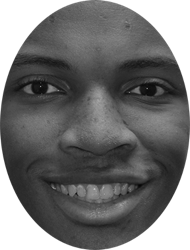 | 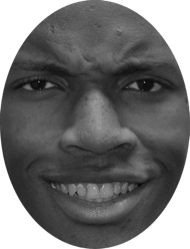 |
| HM09 | 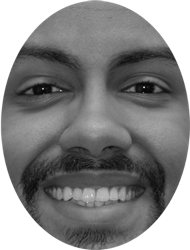 | 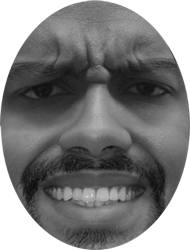 |

**Table S2.**

Percentage of videos recognised by each participant as “familiar” or “somewhat familiar”, during the four experimental conditions that presented a context video (happy or angry)

| Participant No | Percentage of context videos recognized (%) | | | | |
| --- | --- | --- | --- | --- | --- |
|  | Happy context – Happy expression | Happy context – Sarcastic expression | Angry context – Happy expression | Angry context – Sarcastic expression | Across all four conditions |
| 01 | 00 | 00 | 10 | 10 | 05 |
| 02 | 10 | 10 | 50 | 50 | 30 |
| 03 | 40 | 30 | 30 | 40 | 35 |
| 04 | 00 | 00 | 00 | 00 | 00 |
| 05 | 10 | 10 | 00 | 00 | 05 |
| 06 | 00 | 10 | 00 | 20 | 7.5 |
| 07 | 20 | 10 | 20 | 20 | 17.5 |
| 08 | 00 | 00 | 10 | 10 | 05 |
| 09 | 10 | 00 | 10 | 20 | 10 |
| 10 | 30 | 30 | 60 | 90 | 52.5 |
| 11 | 10 | 10 | 20 | 40 | 20 |
| 12 | 20 | 10 | 30 | 60 | 30 |
| 13 | 10 | 20 | 00 | 00 | 7.5 |
| 14 | 20 | 10 | 10 | 40 | 20 |
| 15 | 50 | 30 | 10 | 30 | 30 |
| 16 | 10 | 20 | 10 | 20 | 15 |
| 17 | 20 | 20 | 40 | 10 | 22.5 |
| 18 | 20 | 00 | 00 | 30 | 12.5 |
| 19 | 10 | 00 | 20 | 30 | 15 |
| 21 | 00 | 00 | 10 | 30 | 10 |
| 22 | 10 | 00 | 00 | 00 | 2.5 |
| 24 | 20 | 00 | 20 | 20 | 15 |
| 25 | 20 | 10 | 10 | 30 | 17.5 |
| 26 | 10 | 00 | 00 | 10 | 05 |
| Average | 15 | 10 | 15 | 25 | 16 |

**Table S3.**

Repetitions for each identity presented during all six experimental conditions

CAUCASIAN

| **Identity** | **Repetitions** | **Repetitions per context** | | |
| --- | --- | --- | --- | --- |
|  |  | **No Context** | **Happy Context** | **Angry Context** |
| AF01 | 3 | 1 | 1 | 1 |
| AF06 | 3 | 1 | 1 | 1 |
| AF09 | 3 | 1 | 1 | 1 |
| AF14 | 3 | 1 | 1 | 1 |
| AF16 | 2 | 1 | 0 | 1 |
| AF26 | 3 | 1 | 1 | 1 |
| AF27 | 3 | 1 | 1 | 1 |
| AM07 | 3 | 1 | 1 | 1 |
| AM23 | 3 | 1 | 1 | 1 |
| BF22 | 3 | 1 | 1 | 1 |
| BF29 | 2 | 1 | 1 | 0 |
| BF32 | 2 | 1 | 0 | 1 |
| BM04 | 3 | 1 | 1 | 1 |
| BM05 | 3 | 1 | 1 | 1 |
| BM11 | 3 | 1 | 1 | 1 |
| BM14 | 3 | 1 | 1 | 1 |
| BM21 | 3 | 1 | 1 | 1 |
| BM31 | 3 | 1 | 1 | 1 |
| BM34 | 2 | 1 | 1 | 0 |
| BM35 | 2 | 1 | 1 | 0 |

OTHER RACES

| **Identity** | **Repetitions** | **Repetitions per context** | | |
| --- | --- | --- | --- | --- |
|  |  | **No Context** | **Happy Context** | **Angry Context** |
| AAF05 | 1 | 0 | 0 | 1 |
| AAM09 | 1 | 0 | 0 | 1 |
| HF01 | 2 | 0 | 2 | 0 |
| HM09 | 1 | 0 | 0 | 1 |

**Table S4.**

Bootstrapped 95% confidence intervals (CI) for all participants

| Participant ID | Overall Accuracy | 95% confidence intervals | |
| --- | --- | --- | --- |
|  |  | Upper bound | Lower Bound |
| 01 | 0.88 | 0.78 | 0.95 |
| 02 | 0.70 | 0.58 | 0.82 |
| 03 | 0.91 | 0.84 | 0.97 |
| 04 | 0.65 | 0.53 | 0.78 |
| 05 | 0.87 | 0.78 | 0.95 |
| 06 | 0.75 | 0.63 | 0.85 |
| 07 | 0.73 | 0.61 | 0.83 |
| 08 | 0.83 | 0.73 | 0.92 |
| 09 | 0.77 | 0.65 | 0.87 |
| 10 | 0.73 | 0.62 | 0.83 |
| 11 | 0.98 | 0.95 | 1.00 |
| 12 | 0.85 | 0.75 | 0.93 |
| 13 | 0.87 | 0.80 | 0.95 |
| 14 | 0.87 | 0.77 | 0.95 |
| 15 | 0.70 | 0.59 | 0.81 |
| 16 | 0.82 | 0.72 | 0.92 |
| 17 | 0.92 | 0.85 | 0.98 |
| 18 | 0.83 | 0.73 | 0.92 |
| 19 | 0.78 | 0.68 | 0.88 |
| 21 | 0.82 | 0.72 | 0.92 |
| 22 | 0.78 | 0.67 | 0.88 |
| 24 | 0.95 | 0.89 | 1.00 |
| 25 | 0.92 | 0.83 | 0.98 |
| 26 | 0.92 | 0.86 | 0.98 |
| 27* | 0.60 | 0.48 | 0.72 |

*participant removed from statistical analysis
